# Bile Duct Injury During Cholecystectomy: Necessity to Learn How to Do and Interpret Intraoperative Cholangiography

**DOI:** 10.3389/fmed.2021.637987

**Published:** 2021-02-17

**Authors:** Niki Christou, Alexia Roux-David, David N. Naumann, Stephane Bouvier, Thibaud Rivaille, Sophiane Derbal, Abdelkader Taibi, Anne Fabre, Fabien Fredon, Sylvaine Durand-Fontanier, Denis Valleix, Muriel Mathonnet

**Affiliations:** ^1^Department of Digestive Surgery, University Hospital of Limoges, Limoges, France; ^2^Department of Visceral Surgery, Geneva University Hospitals and Medical School, Geneva, Switzerland; ^3^Department of Surgery, University Hospitals Birmingham NHS Foundation Trust, Birmingham, United Kingdom

**Keywords:** intraoperative cholangiography, interpretation, cholecystectomy, bile duct injury, laparoscopy

## Abstract

**Introduction:** Biliary duct injury (BDI) is a serious complication during cholecystectomy. Perioperative cholangiography (POC) has recently been generating interest in order to prevent BDI. However, the current literature (including randomized controlled trials) cannot conclude whether POC is protective or not against the risk of BDI. The aim of our study was to investigate whether POC could demonstrate earlier BDI and which criteria are required to make that diagnosis.

**Methods:** We performed a retrospective study between 2005 and 2018 in our French tertiary referral center, which included all patients who had presented following BDI during cholecystectomy.

**Results:** Twenty-two patients were included. Nine patients had POC, whereas 13 did not. When executed, POC was interpreted as normal for three patients and abnormal for six. In this latter group, only two cases had a BDI diagnosed intraoperatively. In other cases, the interpretation was not adequate.

**Conclusion:** BDIs are rare but may reduce patients' quality of life. Our study highlights the surgeon's responsibility to learn how to perform and interpret POC in order to diagnose and manage BDIs and potentially avoid catastrophic consequences.

## Introduction

Biliary duct injuries (BDIs) that occur during cholecystectomy (1) are complex complications to manage, both for the patient and the surgical team (2), with potential repercussions for postoperative morbidity and mortality and often significant decrease in quality of life. BDIs encompass cyst duct leakage, accessory bile duct injuries, or common bile duct injuries, with possible injuries on vascular structures especially the right hepatic artery and the portal vein. These injuries can lead to different complications, such as chronic cholangitis and secondary biliary cirrhosis, and potentially the requirement for liver transplantation (3). The incidence of BDI associated with laparoscopy is 0.25–0.74% for “major lesions,” which affect the main bile duct (MBD), the common hepatic duct, and the right hepatic branch as complete section of biliary duct, whereas it is 0.28–1.70% for “minor lesions,” which impact the cystic stump, the cystic duct, and the junction between the cystic duct and the MBD. These figures are higher than those reported after open cholecystectomy ranging from 0.1 to 0.3% (4). Early recognition of the biliary injury, and by extension its prompt management, is directly correlated with the patient's future prognosis (5).

The risk of BDIs may be increased by aberrant anatomy, ignored or misidentified anatomy, difficult pathology, bleeding, thermal injury, inexperience, and overconfidence of the surgeons (6). Different injury prevention strategies exist, especially those that aid in avoiding the misidentification of the MBD by using “the critical view of safety” (7). Furthermore, methods such as subtotal cholecystectomy in case of cholecystitis with hepatic pedicle inflammation, or conversion to open surgery, may reduce the risk (8–11). The role of perioperative cholangiography (POC) and the quality of its interpretation are debated in the context of reducing risk of BDI (12–14). Indeed, the last guidelines from the Society of American Gastrointestinal and Endoscopic Surgeons recommended to use BDI in cases of cholecystitis (present or past), or possible biliary anatomy variations, or intraoperative suspicion of BDI. These recommendations were given with very low certainty of evidence, as the incidence of BDI is very rare, and randomized controlled trials have so far been unable to find differences between surgeries with POC and those without (15).

The current study investigated patients who had a BDI and were referred to our center, in order to highlight the importance of high-quality performance and interpretation of POC in the diagnosis and management of BDI.

## Materials and Methods

This was a retrospective, single-center, observational study including patients treated for a BDI during a cholecystectomy at the tertiary referral center of Limoges University Hospital, Limoges, France, between January 1, 2005, and December 31, 2018. All the methodology was carried out in accordance with relevant guidelines and regulations. The protocol was approved by a named institutional local committee of University Hospital of Limoges. All patients were older than 18 years and were informed of such a study and gave informed consent.

With the collaboration of the Medical Information Department, we have highlighted the records of all patients referred for a BDI during this period, regardless of their origin (peripheral hospital, private hospital, and university hospital) and the initial approach (laparoscopic or open).

All types of iatrogenic injuries were included: minor or more complex, whatever their management (endoscopic, radiological, surgical, or combined). Other biliary injury etiologies, mainly traumatic causes, were excluded.

Minor wounds were defined as those affecting the cystic stump, the cystic duct, and the junction between the cystic duct and the MBD, and major wounds were defined as those affecting the MBD, the common hepatic duct, and the right hepatic branch.

### Data Collection

The following patient data were identified:

- Demographic characteristics [(of which some were patient-related risk factors (RFs)]: sex, age at BDI, body mass index (BMI), (16), a history of an abdominal surgery, a possible source of obstructing cystic pedicle dissection, and the presence of hepatopathy. The origin of the patients (initially at our department or secondarily transferred from a peripheral center) was specified.
- Data relating to cholecystectomy: all these data were noted on the operative report and extracted: the indication (emergency or elective surgery), whether the operation was to be performed as an outpatient; the approach; and the experience of the operator. Surgeons were defined as “junior” with <6 years and “senior” with equal to or more than 6 years' experience. The intraoperative RFs were identified according to the literature on the subject (4, 9, 17) and included the presence of bleeding; the presence of significant local inflammation (adhesions, hepatic pedicle inflammation) or chronic cholecystitis; and the detection of anatomical variations in the termination of the cystic duct or bile ducts (diagnosed intraoperatively or more remotely on the imaging data).
- Data concerning POC: its achievement, its reading and interpretation (normal, incomplete hepatogram, leakage of contrast agent, suspicion of lithiasis in the MBD), and whether it had allowed early diagnosis of a biliary injury.
- Data concerning the BDI: its type, according to the Amsterdam classification (18), specifying its location in relation to biliary convergence; its time of diagnosis [intraoperative, immediate postoperative (before 6 weeks) or late (more than 6 weeks) from the injury]; its mode of discovery (biliary leakage or retention symptoms); and the existence of an associated arterial wound, in particular of the right branch of the hepatic artery. The diagnosis of an arterial wound was made either on the basis of imaging data [injected abdominopelvic computed tomography (CT) scan, more rarely arteriography) or intraoperatively at resumption of surgery. In case of no formal data on the existence of an arterial wound, the diagnosis was made by a radiologist on the basis of the data from the CT scan obtained at arterial time.
- The initial management of the BDI: endoscopic with the performance of endoscopic retrograde cholangiopancreatography (ERCP), whether accompanied by the insertion of a biliary prosthesis or simply the extraction of residual lithiasis. The existence of an unsuccessful attempt to catheterize the MBD was recorded. The initial surgical management was noted: external drainage (by rubber corrugated drains or tube drains in contact with the vesicular bed), choledocholic suture on T-tube (Kehrs), simple suture of the biliary duct in case of a puncture wound, or Roux-en-Y hepaticojejunal anastomosis, whether early (within 6 weeks of the wound) or late (after 6 weeks).
- Late complications linked to the management of the injury: morbidity related to secondary stenosis of a choledocholic suture or biliodigestive anastomosis was considered. The management of complications by radiological, endoscopic, or surgical means was specified. The other complications noted were the presence of a hernia on a laparotomy scar, the presence of acute pancreatitis postsphincterotomy in the case of ERCP, and postoperative hemorrhage requiring emergency reoperation. Mortality was taken into account in the case of patient death in the context of BDI inducing sepsis.

The final follow-up point was the date of the last consultation in digestive surgery at the university hospital or at the original peripheral hospital after the BDI.

After conducting an observational study, the patients were divided into two groups: one group in which they had received POC (POC+) and one group in which POC had not been done (POC–).

### Outcomes

Different outcomes such as the gravity of injuries, the diagnostic time, the delay to surgical treatment, and a composite variable called “morbimortality” encompassing cases of death and anastomotic stenosis were reported.

### Ethics

As this study is a retrospective one without modifying patients' management, defined as “a non-interventional study,” it was approved by the local ethics committee (MR003). Information and right to refusal to patients have been launched.

### Statistical Analysis

The two groups were compared in terms of time to diagnosis, time to management, and postoperative morbidity and mortality using Fisher exact tests. A risk threshold α was determined at 0.05. Odds ratios were also calculated.

## Results

All patients with BDI as defined in section Materials and Methods consecutively presenting to the study institution were included in the analysis; there were 22 patients treated at Limoges University Hospital for a BDI during a cholecystectomy between January 1, 2005, and December 31, 2018. There were no exclusions. No study subjects were lost to follow-up. The demographic characteristics of the patients are summarized in Table 1, with a female-to-male ratio of 1:2. The majority of patients came from outlying centers. Of the seven patients with at least one RF of BDI, four patients had a history of abdominal surgery, and three were obese (BMI >30 kg/m^2^).

**Table 1:** Demographic characteristics.

**Data**	**N (Percentage)**
Sex-men	10 (45.5%)
Mean age (years)	61.7 ± 17.3
**Origin**	
CHU	4 (18.2%)
Clinic	2 (9.1%)
Peripheric hospital (CH) center	16 (72.7%)
**PRF of BDI**	
None	14 (63.6%)
1	7 (31.8%)
≥2	1 (4.6%)
**Average follow-up time**	**In months**
From the BDI	14.5
Since the biliary repair	12.6

*CHU, university hospital center; CH, hospital center; PRF, personal risk factor (obesity, history of abdominal surgery, hepatopathy)*.

Table 2 summarizes the data for cholecystectomy. All patients who had emergency surgery had acute cholecystitis. Among those who had elective surgery, the majority had “complicated” vesicular lithiasis (numerous interventions at a distance from the acute episode of cholecystitis or chronic cholecystitis). In 81.8% of patients (18 patients), at least one intraoperative RF for BDI was found, and among these, at least two RFs were found in 55.6% of patients. Patients who had emergency surgery were at greater risk of having pedicle hepatic inflammation or intraoperative bleeding. Patients who had elective surgery (interventions at a distance from the acute episode of cholecystitis for the most part) had more chronic cholecystitis or cholecystitis fistulas (two duodenal fistulas and one antropyloric fistula). Cholangiography was performed in nine cases (40.9% of patients), more often as part of scheduled surgery than as an emergency.

**Table 2:** Data of the cholecystectomy.

**Data**	***N* (Percentage)**
Surgery conditions - elective	13 (59.1%)
Operative indications	
History of complicated vesicular lithiasis	9 (40.9%)
Present acute lithiasis cholecystitis	9 (40.9%)
**Surgical approach**	
Laparoscopy	17 (77.3%)
Converted laparoscopy	3 (13.6%)
Surgeon **-** senior	20 (90.9%)
**Intra operative difficulties**	
Local inflammation/hepatic pedicle inflammation	12 (54.5%)
Chronic Cholecystitis	8 (36.4%)
Anatomical variations	4 (18.2%)
Cholecystodigestive fistula	4 (18.2%)
Intraoperative bleeding	3 (13.6%)
Voluminous left lobe / Biliary cyst	1 (4.5%)
Realisation of a cholangiography	9 (40.9%)
Intra operative diagnosis of BDI	5 (22.7%)

Table 3 indicates the characteristics of the BDIs included in this study before their arrival at Limoges Center. The injuries were divided into four types according to the Amsterdam classification (Appendix 1). There were two patients with a type I injury, six with a type IV injury, five with an injury linked to a surgical clip positioned on the MBD, seven patients with a lateral BDI, and two patients with a stone found in the MBD. Wound management was often multidisciplinary with the exception of “minor” wounds of the cystic duct or accessory vesicular canal. By taking into account each treatment, 20 patients benefited from surgery, 17 had an endoscopy, and 7 underwent a radiologic treatment. More precisely, focusing on the patients, there was an association with endoscopic management in 68.2% of cases (15 patients) and with radiological management in 18.2% of cases (four patients). Endoscopic management, when performed, consisted of failed catheterization of the MBD in 66.7% of cases (10 patients, with either a complete section or MBD stenosis), placement of MBD prosthesis in 26.7% of cases (four patients, all with a lateral wound), and simple endoscopic sphincterotomy for residual MBD lithiasis in 6.6% of cases (one patient). Interventional radiology facilitated the drainage of bilioma or abscess in 50% of the cases (two patients), transcutaneous drainage of the bile ducts preoperatively after failure of endoscopic drainage in 25% of the cases (one patient), and the performance of these two gestures in 25% of the patients (one patient).

**Table 3:** Data linked to the biliary duct injury.

**Data linked to the BDI**	
**Characteristics**	***N*** **=** **(Percentage)**
**Relationship to biliary convergence**	
Injury of convergence or proximal	12 (54.5%)
**Type of wound**	
Lateral MBD injury	7 (31.8%)
Complete Section MBD	6 (27.3%)
MBD Clips	5 (22.7%)
Secondary necrosis of MBD	2 (9.1%)
Accessory conduit leak (cystic, Luschka)	2 (9.1%)
**HA right branch associated lesion**	
Yes	9
**Diagnostic time**	
*Intra* operative	5 (22,7%)
*Post-operative* immediate (<6weeks)	14 (63,6%)
**Associated endoscopic treatment, of which:**	15 (68,2%)
Endoscopic sphincterotomy alone	1 (6,6%)
MBD Prosthesis	4 (26,7%)
MBD catheterization failure	10 (66,7%)
**Associated endoscopic support, including**	4 (18,2%)
Trans-hepatic biliary drainage	1 (25%)
Both	1 (25%)
**Initial surgical management, including**	21 (95,4%)
External drainage only	1 (4,8%)
External drainage before repair surgery	5 (23,8%)
Choledocholic suture on T-tube	3 (14,3%)
Simple Suture	3 (14,3%)
BDA: early <6 weeks post-operatively	5 (23,8%)
BDA: late > 6 weeks post-operatively	9 (42,8%)
HA: Hepatic Artery MBD: Main Bile Duct BDA: Bilio-digestive anastomosis	

Further surgery was required for almost all patients (95.4%). Strategies included repair between the two segments of the injured MBD, a biliodigestive anastomosis within variable delays, or more rarely a simple suture of an accessory duct. Table 4 specifies these surgical interventions. Patients underwent right subcostal laparotomy in 90.5% of cases (19 patients) and laparoscopy in 9.5% of cases (two patients for lavage, drainage, and suture of a cystic duct in one case or a punctiform bile duct wound in the second case). Only one patient died before any surgical reoperation.

**Table 4:** Characteristics of biliary injuries by type according to the Amsterdam classification.

**Characteristics**	**Type I *N* = 2 (9.1%)**	**Type II *N* = 7 (31.8%)**	**Type III *N* = 5 (22.7%)**	**Type IV *N* = 8 (36.4%)**	**Total (*N* = 22)**
**Injuries types**					
Injuries distally to the BC	2 (100%)	4 (57.1%)	1 (20.0%)	5 (62.5%)	12 (54.5%)
**HA right branch associated lesion**					
Yes	0 (0%)	3 (42.9%)	2 (40.0%)	4 (50%)	9 (40.9%)
**Diagnostic time**					
*Intra* operative	0 (0%)	2 (28.6%)	0 (0%)	3 (37.5%)	5 (22.7%)
*Post-operative* immediate (<6 weeks)	2 (100%)	5 (71.4%)	2 (40.0%)	5 (62.5%)	14 (63.6%)
**Discovery mode**					
Biliary leakage	2 (100%)	5 (71.4%)	0 (0%)	8 (100%)	15 (68.2%)
Biliary retention	0 (0%)	2 (28.6%)	5 (100%)	0 (0%)	7 (31.8%)
Associated endoscopic treatment, of which:					15 (68,2%)
Endoscopic sphincterotomy alone	0 (0%)	0 (0%)	1 (20.0%)	0 (0%)	1 (6.6%)
MBD Prosthesis	0 (0%)	3 (42.9%)	1 (20.0%)	0 (0%)	4 (26.7%)
MBD catheterization failure	0 (0%)	2 (28.6%)	2 (40.0%)	6 (75.0%)	10 (66.7%)
Associated radiologic treatment, of which:					4 (18,2%)
Biliome/abcess drainage	0 (0%)	2 (28.6%)	0 (0%)	0 (0%)	2 (50%)
Transhepatic biliary drainage	0 (0%)	0 (0%)	0 (0%)	1 (12.5%)	1 (25%)
Both	0 (0%)	0 (0%)	0 (0%)	1 (12.5%)	1 (25%)
Initial surgical treatment, of which:					21 (95,5%)
External drainage only	0 (0%)	1 (14.3%)	0 (0%)	0 (0%)	1 (4.8%)
External drainage before surgery	0 (0%)	2 (28.6%)	1 (20.0%)	2 (25.0%)	5 (23.8%)
Choledocholic suture on T-tube	0 (0%)	1 (14.3%)	0 (0%)	2 (25.0%)	3 (14.3%)
Simple suture	2 (100.0%)	1 (14.3%)	0 (0%)	0 (0%)	3 (14.3%)
BDA early <6 weeks post-operatively	0 (0%)	2 (28.6%)	0 (0%)	3 (37.5%)	5 (23.8%)
**Morbi-mortality**					
Anastomotic Stenosis	0 (0%)	3 (42.9%)	1 (20.0%)	4 (50%)	8 (36.4%)
Death	0 (0%)	1 (14.3%)	0 (0%)	1 (14.3%)	2 (9.1%)

Patients' mean follow-up was 14.5 months from the BDI and 12.6 months postoperatively in the operated patients' subgroup (95.4% or 21 patients). During the follow-up, 9.1% of patients (two patients) died of complications directly attributable to the BDI (sepsis resistant to any treatment). All patients initially treated with a choledocholic suture on T-tube (13.6% of patients) developed anastomosis stenosis. In all the cases, surgical management with biliodigestive anastomosis was required. One-third of the patients operated on for BDI by biliary–biliary or biliary–digestive anastomosis required an additional procedure, either surgical or radiological, to treat an anastomotic stenosis during follow-up. None of the patients treated with simple cystic suture or puncture wound suture required further management within the limits of follow-up. Other complications included acute pancreatitis after ERCP in one patient, right subcostal hernia in patients reoperated on by laparotomy (two patients), and hemorrhage requiring an additional operation for hemostasis in one patient.

Concerning the role of cholangiography performed during initial surgery (Table 5), it was performed in 40.9% of patients in the series (nine patients) and interpreted as normal in 33.3% of cases (three patients) and abnormal in 66.7% of cases (six patients). An incomplete hepatogram was found in five patients and a suspicion of residual lithiasis in the MBD in one case. In patients with a cholangiogram considered as abnormal (incomplete hepatogram), the BDI was diagnosed intraoperatively in only two patients. In the other cases, according to the operative report, the surgeon interpreted the incomplete hepatogram as a “problem of leakage in the cystic duct” or, in some cases, was “sure of his technique and did not explore this abnormality further.” The remaining 66.7% of wounds with abnormal POC were diagnosed immediately postoperatively (<6 weeks) with, in order of frequency (most to the least), abdominal pain, jaundice, and biliary peritonitis. BDIs in these patients who had undergone cholangiography, regardless of its interpretation, were predominantly distally to the biliary convergence in two-thirds of cases.

**Table 5:** Perioperative cholangiography and biliary duct injury.

	**POC**			**BDI**			
**Patients**	**Performed**	**Results**	**BDI diagnosis with POC**	**TYPE of BDI**	**Discovery mode**	**Delay of discovery**	**Associated Arterial wound**
Patient N°1	No	NA	No	L-BDI	L	PO	Missing Data
Patient N°2	No	NA	No	CLIP	R	PO-I	0
Patient N°3	Yes	IH	No	D	L	PO-I	1
Patient N°4	No	NA	No	CLIP	R	PO-L	Missing Data
Patient N°5	No	NA	No	D	L	PO	Missing Data
Patient N°6	No	NA	No	L-BDI	R	PO	No
Patient N°7	Yes	IH	No	SN	L	PO-I	No
Patient N°8	Failure	NA	No	CLIP	R	PO-I	Yes
Patient N°9	Yes	S	No	D	L	PO-I	Missing Data
Patient N°10	No	NA	No	L-BDI	R	PO-I	Missing Data
Patient N°11	Yes	N	No	SN	L	PO-I	Missing Data
Patient N°12	Yes	IH	Yes	D	L	PO	Yes
Patient N°13	No	NA	No	CLIP	R	PO-L	Missing Data
Patient N°14	No	NA	No	L-BDI	L	PO-I	Missing Data
Patient N°15	No	NA	No	CLIP	R	PO-L	No
Patient N°16	No	NA	No	L-BDI	L	PO-I	Missing Data
Patient N°17	Yes	IH	Yes	D	L	PO	Yes
Patient N°18	Yes	IH	No	L-BDI	L	PO-I	No
Patient N°19	No	NA	No	D	L	PO-I	Missing Data
Patient N°20	No	NA	No	L-BDI	L	PO-I	Yes
Patient N°21	Yes	N	No	A	L	PO-I	No
Patient N°22	Yes	N	No	A	L	PO-I	No

*Results: N, Normal; IH, incomplete hepato-gram; S, “Stone” /Lithiasis in the Main Bile duct; NA, non-achieved*.

*Type of BDI: A, Injury Type A of Amsterdam: Leakage on the cystic duct/ accessory canal; L-BDI, lateral BDI; D, injury type D of Amsterdam including only Comlete section Main Bile Duct*.

*CLIP, CLIP MBD; SN, secondary necrosis*.

*Discovery mode: L, biliary leakage; R, biliary retention*.

*Delay of discovery: PO, per operative; PO-I, post-operative immediate <6 weeks; PO-L, post-operative late> 6 weeks*.

POC was not performed in 59.1% of cases (13 patients); the BDIs were diagnosed intraoperatively in 23.1% of cases (bile leakage in the operating field), immediately postoperatively in 53.8% of cases, and late postoperatively in 23.1% of cases. The majority of these were distal-convergence injuries (53.8%), but with a higher percentage of complex/proximal convergence injuries than the cholangiography group (46.2%).

When comparing the two groups of patients (Tables 6, 7A,B) (POC vs. no POC), there were no significant differences in the severity of the lesions, the time of BDI diagnosis, or the delay of surgical treatment and morbidity or mortality. The ORs of morbidity or anastomotic stenosis were 2.24 and 2.85, respectively.

**Table 6:** Impact of intraoperative cholangiography in the management of biliary duct injury.

**Variables**	**Without POC *N* = 13 (59.1%)**	**With POC *N* = 9 (40.9%)**	**OR [IC95%] (*p-*value)**
Injuries gravity			
Injuries distally to the BC	7 (53.8%)	6 (66.7%)	0.59 [0.07–4.52] (*p* = 0.67)
Complex injuries	6 (46.2%)	3 (33.3%)	1.67 [0.22–15.12] (*p* = 0.67)
Diagnostic Time			
*Intra* operative	3 (23.1%)	3 (33.3%)	0.61 [0.06: 6.16] (*p* = 0.65)
*Post-operative* immediate <6 weeks	7 (53.8%)	6 (66.7%)	0.59 [0.07 : 4.52] (*p* = 0.67)
*Post-operative* late> 6 weeks	3 (23.1%)	0 (0%)	∞ [0.29: ∞] (*p* = 0.24)
Delay to surgical treatment			
*Intra* operative	2 (15.4%)	1 (11.1%)	1.4 [0,06: 96,32] (*p* = 1)
*Post-operative* immediate <6 weeks	3 (23.1%)	5 (55.6%)	0.26 [0,03: 2,07] (*p* = 0.19)
*Post-operative* late > 6 weeks	6 (46.1%)	3 (33.3%)	1.67 [0,22: 15,12] (*p* = 0.67)
Failure of late reparation	1 (7.7%)	0 (0%)	∞ [0,02 : ∞] (*p* = 1)
Death before surgery	1 (7.7%)	0 (0%)	∞ [0,02 : ∞] (*p* = 1)
Morbi-mortality	7(53.8%)	3 (33.3%)	2,24 [0,30: 20,39] (*p* = 0.41)
Anastomotic Stenosis	6 (46.1 %)	2 (22.2%)	2,85 [0.34-38.66] (*p* = 0.38%)
Death	1 (7.7%)	1 (11.1%)	0.68 [0.01 - 58.78] (p=1)
OR: Odds Ratio[….] Interval of confidence at 95%			

**Table 7A:** Demographic characteristics of patients' group with POC performed.

**Patients' characteristics**					**POC**	**Cholecystectomy**				**Follow-up time**
**Patient number**	**Age when BDI**	**Sex**	**Risk factors for bdi**	**Origin**	**Performed**	**Surgery condition**	**Type of surgery**	**Surgeon's experience**	**Per operative difficulty**	
3	46 (2015)	M	0	1	Yes	1	1	1	2	1
7	82 (2015)	M	0	3	Yes	2	1	2	3 + 2	2
8	36 (2016)	F	0	2	Failure	2	1	2	0	1
9	51 (2011)	M	2	2	Yes	1	2	2	3+4	1
11	69 (2010)	M	0	2	Yes	2	1	2	1+3	1
12	83 (2013)	F	0	2	Yes	2	1	2	2+3	2
17	71 (2017)	M	0	2	Yes	1	1	2	2	1
18	51 (2018)	F	1	1	Yes	2	1	1	0	1
21	78 (2018)	F	1	1	Yes	2	2	2	0	2
22	47 (2018)	F	1	1	Yes	2	1	2	0	1
			0 No	1 University Hospital Of Limoges		1 Emergency	1 Laparoscopy	1 Junior	0 No	
			1: 1 Risk factor	2 Peripheric hospital		2 Elective	2 OPEN	2 SENIOR	1 Anatomical variations	
			2: ≥2 RISK FACTORS	3 Private Hospital			3 CONVERSION TO OPEN		2 Local inflammation	
									3 Chronic cholecystitis	
									4 Intra operative bleeding	
									5 Large left liver or cyst in the liver	

**Table 7B:** Demographic characteristics of patients' group without POC performed+.

**Patients' characteristics**					**POC**	**Cholecystectomy**				**Follow-up time**
**Patient number**	**Age when BDI**	**Sex**	**Risk factors for BDI**	**Origin**	**Non-performed**	**Surgery condition**	**Type of surgery**	**Surgeon's experience**	**Per operative difficulty**	
1	29 (2012)	F	0	3	No	2	1	2	2 + 3 + 1	1
2	68 (2013)	F	0	2	No	2	1	2	2	1
4	46 (2012)	M	0	2	No	2	1	2	1	2
5	54 (2013)	F	0	2	No	1	3	2	2 +4	2
6	85 (2010)	M	1	2	No	1	3	2	1+2+5	2
10	71 (2011)	M	0	2	No	1	1	2	2+3+4	2
13	39 (2011)	F	1	2	No	1	1	2	1	1
14	77 (2010)	M	0	2	No	2	3	2	2+3	1
15	45 (2016)	F	0	2	No	2	1	2	0	1
16	90 (2016)	F	1	2	No	1	1	2	2+3+1	1
19	70 (2017)	M	0	2	No	1	1	2	2	2
20	69 (2018)	F	1	2	No	2	1	2	2	1
			0 NO	1 University hospital of limoges		1 Emergency	1 Laparoscopy	1 Junior	0 NO	
			1: 1 risk factor	2 Peripheric Hospital		2 Elective	2 Open	2 Senior	1 Anatomical variations	
			2: ≥2 risk factors	3 Private Hospital			3 Conversion to open		2 Local inflammation	
									3 Chronic cholecystitis	
									4 Intra operative bleeding	
									5 Large left liver or cyst in the liver	

## Discussion

Despite non-significant results in a small population, our retrospective study in a small but homogeneous cohort of patients suggests that POC reduced the severity of biliary tract wounds, time to management, and the risk of long-term stenosis.

Several studies that have examined BDI and laparoscopic cholecystectomy have found major BDI rates of 0.15–0.36% and an overall biliary complication rate of 1.5% if bile leaks are included (1). The value of the POC is still being debated.

The review of Slim et al. (15), including six comparative large-scale studies, demonstrated conflicting results. Ludwig et al. (13) in their meta-analysis in 2002, reported a protective effect of intraoperative cholangiogram on BDI with 87% diagnosis of BDI at the time of cholecystectomy (much higher than our current cohort). However, Nuzzo et al. (19), in their Italian multicentric retrospective study with more than 56,000 patients, pointed out no difference in incidence and intraoperative detection of BDI with routine cholangiography, a finding that was echoed by Giger et al. (20) a Swiss retrospective multicentric study (2011).

To demonstrate efficacy of POC, between 12,000 (21) and 26,000 patients (22) are needed in a prospective comparative study with a power between 80% and 90%. Despite our non-significant results, there is a certain profile of patients with more severe BDI and higher postoperative morbidity and mortality in the group without POC.

Our main aim was to focus on patients with a BDI, highlighting the performance of POC and the role of the individual surgeons. The utilization of POC is part of an atmosphere of risk prevention, in which both the surgeon and the whole operating theater team participate. Here we illustrate that a BDI can occur in multiple scenarios across a spectrum, including during “simple” cholecystectomies performed by a trained operator (59.1% of the wounds in this study were made during scheduled surgery). This is in keeping with the literature, which emphasizes that adequate training for cholecystectomy is mandatory but does not prevent all injuries at all times (23). Moreover, a large number of BDIs occur in surgeries considered as more straightforward (19, 24). The behaviors and the attention of both the surgeon and the surgical team are important. The surgeon must select patients carefully, taking into account both surgical and patients' related RFs in a patient-centered manner. Moreover, the surgeon must be familiar with all anatomical variations and surgical techniques (such as open vs. laparoscopy). This is especially the case as anatomical variations can cause misperceptions and errors that lead to false reassurance, resulting in BDIs (4, 25).

Anatomical variations in the Calot's triangle are frequent. Thus, in the review of Abdalla et al. concerning the Calot's triangle anatomy, sometimes referred to as the cystohepatic or hepatocystic triangle, the variations may concern the origin and course of the cystic artery or the ductal system. In only 75% of cases the cystic artery is regular and originates from the right hepatic artery. Accessory biliary ducts could been found in 1–30% of patients (26). If these ducts are injured during manipulation of Calot's triangle, there may be serious biliary leakage.

Moreover, even if laparoscopy is an ever-increasing technique, the incidence of BDIs is still higher than with open surgery. This highlights the importance of surgeons' familiarity with the open technique and the optimal timing for conversion when required (27), as well as the utility of asking for another surgeon opinion (28) to decrease the risk of misperceptions.

POC may be one of the various means of preventing BDI, and the latest recommendation from the Prevention of Bile Duct Injury Consensus Work Group (1) is that POC leads to “early recognition and avoidance of potentially increasing the severity of BDI.” However, its performance alone is not enough; its interpretation is crucial. We have seen in this study that even abnormal POCs, even if they are found to be abnormal by the surgeon, are not always enough to diagnose an injury. It may be prudent therefore to train young surgeons to carry out this procedure systematically as soon as possible and above all to interpret it meticulously. It can be a simple, minimally invasive procedure that may be of great service.

From a methodological point of view, the study has several biases. Because of its retrospective nature, the items considered in determining RFs for preoperative or intraoperative biliary injuries depended on the surgeons' experience and their own intraoperative assessment of the operation. Patients came from different centers, with difficult access to cholangiography for some. The mean follow-up was 12.6 months after biliary repair, which is too short to assess the risk of stenosis that may occur in the first 2 or 3 years (29). For Navez et al. (30) the median time to onset of biliary stenosis was even 154 months in a cohort of 120 patients. It is therefore uncertain whether some patients may have consulted another center for a later problem and were not included in our study cohort.

We have set the limit of the surgeon's experience at 6 years in accordance with Schwaitzberg et al.'s study (31), where it was shown that more experienced surgeons with an average of 20.7 years of surgical experience had a lower BDI rate than those with approximately 6.1 years of practice (i.e., physicians in training).

On the contrary, our study presents different positive aspects such as longitudinal follow-up and reporting of intraoperative findings. In addition, we can highlight a specific strength of our study, which is lacking from larger studies. Indeed, as previously mentioned, there was poor accuracy of intraoperative cholangiogram interpretation among surgeons; of six subjects who had abnormal POC, intraoperative BDI was diagnosed in only two subjects. This result is in accordance with the study of Sanjay et al. (12), who reported the same observation.

## Conclusion

BDIs are a serious complication of cholecystectomy, which is the most commonly performed procedure in visceral surgery (approximately 100,000 cholecystectomy per year in France). Despite the low incidence of BDI, they are highly significant because of the important longer-term effect on both prognosis and quality of life of patients. While there are identifiable patient-related and intraoperative RFs, BDIs can still occur at the end of a “simple” cholecystectomy, and no surgeon is immune from risk. Intraoperative cholangiography may be a simple way to avoid BDI and mitigate its consequences by reducing the time to diagnosis and management. High-quality POC requires a knowledge of the technique, optimal safety conditions, and competent interpret of the images, which depends on the multiple factors such as surgical training, team dynamics, and operating room environment.

## Data Availability Statement

The raw data supporting the conclusions of this article will be made available by the authors, without undue reservation.

## Ethics Statement

The studies involving human participants were reviewed and approved by Local Ethics Committee of University Hospital Limoges, France. The patients/participants provided their written informed consent to participate in this study.

## Author Contributions

AR-D and MM conceived and designed the study. AR-D acquired the data. AR-D and NC analyzed the data. AR-D, MM, DN, and NC interpreted the data. AR, NC, DN, and MM contributed to the writing of the manuscript and to its critical revision, NC, AR-D, DN, AT, FF, SB, AF, TR, SD, SD-F, DV, and MM approved the final version of the manuscript.

## Conflict of Interest

The authors declare that the research was conducted in the absence of any commercial or financial relationships that could be construed as a potential conflict of interest.

## Abbreviations

BDI: bile duct injury
POC: perioperative cholangiography.

## Appendix

Classification of Amsterdam.

- Type A: leakage in the cystic duct.
- Type B: leakage on the main bile duct (MBD).
- Type C: stenosis or ligation of the MBD.
- Type D: transection of the MBD or one of the main hepatic ducts.
